# Psychedelics in the treatment of unipolar and bipolar depression

**DOI:** 10.1186/s40345-022-00265-5

**Published:** 2022-07-05

**Authors:** Oliver G. Bosch, Simon Halm, Erich Seifritz

**Affiliations:** Department of Psychiatry, Psychotherapy and Psychosomatics, Psychiatric University Hospital Zurich, University of Zurich, Lenggstrasse 31, PO Box 1931, 8032 Zurich, Switzerland

## Abstract

This is a narrative review about the role of classic and two atypical psychedelics in the treatment of unipolar and bipolar depression. Since the 1990s, psychedelics experience a renaissance in biomedical research. The so-called classic psychedelics include lysergic acid diethylamide (LSD), psilocybin, mescaline and ayahuasca. Characteristic effects like alterations in sensory perception, as well as emotion- and self-processing are induced by stimulation of serotonin 2A receptors in cortical areas. The new paradigm of psychedelic-assisted psychotherapy suggests a therapeutic framework in which a safely conducted psychedelic experience is integrated into a continuous psychotherapeutic process. First randomized, controlled trials with psilocybin show promising efficacy, tolerability, and adherence in the treatment of unipolar depression. On the other hand, classic psychedelics seem to be associated with the induction of mania, which is an important issue to consider for the design of research and clinical protocols. So called atypical psychedelics are a heterogeneous group with overlapping subjective effects but different neurobiological mechanisms. Two examples of therapeutic value in psychiatry are 3,4-methyl​enedioxy​methamphetamine (MDMA) and ketamine. Since 2020 the ketamine enantiomer esketamine has been granted international approval for treatment-resistant unipolar depression, and also first evidence exists for the therapeutic efficacy of ketamine in bipolar depression. Whether psychedelics will fulfil current expectations and find their way into broader clinical use will depend on future rigorous clinical trials with larger sample sizes. A well-considered therapeutic and legal framework will be crucial for these substances to create new treatment settings and a potential paradigm shift.

## Introduction

The discovery of lysergic acid diethylamide (LSD) by the Swiss chemist Albert Hofmann in 1943 triggered a worldwide interest into the substance’s unique effects on the human psyche and its potential clinical use and value for research (Nichols and Walter, 2021). Challenging the existing paradigms of psychiatry and psychotherapy, LSD promised to be a tool to better understand the human mind and mental disorders. It is widely seen as the prototype of a psychedelic drug. The term *psychedelic* itself was introduced by the British psychiatrist Humphry Osmond combining the Greek words ψυχη (psychḗ, *soul*) and δῆλος (dẽlos, *to make visible, to reveal*) hence meaning *mind revealing* or *mind manifesting* (Osmond, 1957). Only some years later the writer and philosopher Aldous Huxley popularized the term in his culturally influential essay *The Doors of Perception,* in which he described his subjective experience with the phenylalkylamine mescaline, another classic psychedelic (Huxley, 1959). Following Hofmann’s discovery of LSD, his employer, the Swiss pharmaceutical company Sandoz, enabled an extensive research effort by distributing the substance under the name *Delysid* to researchers around the world. During this first wave of psychedelic research over a time of 15 to 20 years, psychiatrists investigated the substance’s therapeutic potential in the treatment of numerous psychiatric conditions including affective disorders, substance use and chronic pain (Grispoon and Bakalar, 1981; Nichols and Walter, 2021). These studies included more than 40′000 subjects, showing often promising improvement rates but also methodological flaws such as lack of blinding and control groups. A recent analysis of 19 of these early studies showed improvement in depressive symptoms in 79.2% of the 423 Patients after administration of a psychedelic (Rucker et al., 2016). The 1960s saw an increasing interest into psychedelics not only in science but also in the *counterculture* movement, culminating in an increasing idealization and controversy as well as instrumentalization from both its advocates and opponents (Dyck, 2005). The result of this dispute was a sudden interruption of research after the international prohibition of LSD in 1970 (UN, 1971). Subsequently, the discoverer Hofmann himself in hindsight called the chemical his *problem child* (Hofmann, 2012).

Nonetheless, this problem child emerged to an initially subtle but soon rapidly accelerating renaissance starting in the early 1990s, which showed a renewed scientific and later clinical engagement with psychedelic substances (Cameron and Olson, 2018). Simultaneously, scientists started to investigate several other molecules with partly overlapping subjective effects, but different neurobiological mechanisms. This heterogeneous group of so-called atypical psychedelics such as 3,4-methyl​enedioxy​methamphetamine (MDMA) and ketamine soon also showed potential therapeutic effects in psychiatry (Vollenweider, 2001). In a parallel development, new advances in neuroimaging techniques such as positron emission tomography, single-photon emission computed tomography and functional magnet resonance imaging facilitated a new and differentiated understanding of psychedelic-induced altered states of consciousness (Vollenweider et al., 1997; Hermle et al., 1992). Finally, two further developments set up the stage for the definitive comeback of psychedelic substances into laboratories and clinics: on the one hand the increasing neuroscientific investigation of psychotherapeutic processes and on the other hand the so called *psychopharmacology in crisis* (Nutt and Goodwin, 2011), i.e. the withdrawal of the pharmaceutical industry from psychiatric drug development. In the following article, we will describe the most important psychedelics, their subjective effects, and mechanisms of action. We also will give an overview of current evidence on psychedelics in the treatment of unipolar and bipolar depression and potential future challenges. This is a narrative review, for which we performed a Pubmed search using the terms “psychedelic”, “LSD”, “psilocybin”, “mescaline”, “ayahuasca”, “DMT”, “ketamine”, “MDMA”, together with “depression”, “bipolar disorder”, “mania”, “unipolar depression”, “bipolar depression”, “pharmacokinetic”, “pharmacology”, “subjective effects”, “neurobiology”, “neuropsychopharmacology”, “mechanisms”, “psychotherapy”. Then, relevant information was extracted regarding our section topics and included accordingly.

## Psychedelic substances

Psychedelics can be separated into classic and atypical (Calvey and Howells, 2018). Classic psychedelics (sometimes referred to as hallucinogens) are a group of psychoactive substances inducing a characteristic psychedelic experience with distinct changes in consciousness, visual perception as well as emotional, social- and self-processing. Despite the complexity of their pharmacological mechanisms, compelling evidence suggests that serotonergic neurotransmission—especially the serotonin 2A receptor—plays a key role in the mediation of their effects. Unsurprisingly, some of these molecules show structural similarity to serotonin itself (Vollenweider and Kometer, 2010). Classic psychedelics can be further distinguished into three classes: tryptamine derivates (such as psilocybin and N,N-dimethyltryptamine [DMT]), phenylalkylamines such as mescaline, and ergoline derivatives such as LSD. The relatively short history of psychedelics in the western world began with early research on mescaline in the early twentieth century and was then accelerated by the discovery of LSD by Hofmann in 1943 (Nichols and Walter, 2021). On the contrary, in Central- and South America plant-derived substances like psilocybin, DMT (in the form of ayahuasca) and mescaline (in the form of peyote and the san pedro cactus) look back on a rich history in traditional medicinal and spiritual use of 1´000 to over 5′000 years (Miller et al., 2019, Bruhn et al., 2002, Carod-Artal, 2015, Carod-Artal and Vazquez-Cabrera, 2006). The term atypical psychedelics can be used to summarize substances that also induce characteristic altered states of consciousness and profound perceptual changes but do so via various molecular mechanisms and pharmacological receptor profiles (Calvey and Howells, 2018). Two examples of therapeutic value in psychiatry are the entactogen MDMA and the dissociative anesthetic ketamine (and its enantiomer esketamine).

### LSD, etc.

LSD is a semisynthetic derivative of lysergic acid, which naturally occurs in ergot fungi such as *Claviceps purpurea,* growing parasitically on rye and other types of grass (Passie et al., 2008). Chemically it is an indole alkaloid with a chiral structure and with only the d-isomer being psychoactive. Pharmacodynamically, it shows a diversified profile primarily acting as a partial agonist of serotonin 2A and 1A receptors, but also of other serotonergic as well as dopaminergic D1 and D2 and adrenergic receptors. Pharmacokinetically, LSD has a high potency: the lower threshold for light subjective effects lies at approximately 5 µg p.o. (approx. 0.35 µg/kg)(Hutten et al., 2020), while the common dose to induce a full psychedelic effect is usually set between 100 and 200 µg p.o. (approx. 1.4 bis 2.8 µg/kg) (Dolder et al., 2017). The duration of these effects is dose-dependent and reported between 6 and 11 h with possibly longer lasting aftereffects. The LD_50_ is varying between species between 0.3 and 60 mg/kg i.v., and very rare overdose-related deaths in humans were mostly linked to suicide or accidents after increased risk-taking under influence of the drug (Passie et al., 2008; Schlag et al., 2022). However, also three cases with potential toxicity-linked deaths were reported (Nichols and Grob, 2018).

### Psilocybin

Psilocybin (4-phosphoryloxy-N,N-dimethyltryptamine) is a naturally occurring alkaloid with a tryptamine structure containing an indole ring and ethylamine substitute (Passie et al., 2002). It is found around the globe in over 200 fungal species, mostly of the genus *Psilocybe*, which are also referred to as *Magic Mushrooms.* Ceremonial and spiritual human use of these psychoactive mushrooms has a long tradition in indigenous cultures especially in South- and Central America, where the mushrooms are regarded as sacred by the Mazatec people (Miller et al., 2019, Diaz, 1977, Wasson, 1980). Psilocybin is a prodrug that is dephosphorylated into its active compound psilocin which binds with high affinity to serotonin 2A and with lower affinity to other serotonergic receptors. It is effective in a dose range of approx. 20 to 40 mg p.o. (approx. 0.3 to 0.6 mg/kg) with effects lasting about 3 to 6 h (Brown et al., 2017a). The LD_50_ in mice is 280 mg/kg, and very rare overdose-related deaths in humans were mostly linked to accidents after increased risk-taking under influence of the drug (Passie et al., 2002; Schlag et al., 2022).

### Mescaline

Mescaline (3,4,5-trimethoxyphenethylamine) is a plant derived phenylethylamine, which naturally occurs in different cacti species such as the Central American peyote-cactus or *Lophophora williamsii* and the South American san pedro cactus or *Trichocereus macrogonus* var. *pachanoi* (Dinis-Oliveira et al., 2019)*.* Human ritual use of peyote dates back 5′700 years and today mescaline is one of the few psychedelics that can be legally used for religious purposes in the USA (Bruhn et al., 2002). Chemically, it shows structural similarities to the synthetic atypical psychedelic MDMA. Contrary to other classic psychedelics, mescaline mainly acts as a serotonin 2C agonist and shows a lower affinity to 2A receptors. Consequently, it shows a lower psychedelic potency with doses of 200 to 400 mg (approx. 2.8 to 5.7 mg/kg) required for the full effect. With 10 to 12 h, it has a rather long duration of effect. The LD_50_ varies between species (50 to 157 mg/kg i.v.) and there are no reported lethal overdoses in humans (Dinis-Oliveira et al., 2019; Schlag et al., 2022).

### Ayahuasca

Ayahuasca is the commonly used name for a plant-based brew that has been used in religious rituals in indigenous communities in the amazon region for centuries, with recent findings showing the ritual use of ayahuasca ingredients in South America for at least 1′000 years (Miller et al., 2019). Its psychedelic properties are determined by the combination of the classic psychedelic DMT, which is commonly present in *Psychotria viridis,* and monoaminoxidase inhibitors (MAO), such as harmaline and harmine, derived from the liana *Banisteriopsis caapi* (Hamill et al., 2019). The combination is necessary to prevent DMT from being degraded by MAO-enzymes in the gastrointestinal-tract, when taken orally. The tryptamine-based molecule of DMT shows structural similarities to psilocybin and serotonin and the strong psychological effect is mediated through serotonin 2A and 2C receptor agonism. Analysis of samples taken from the amazon region show a wide spectrum of potential dose combinations in the used plants, where nine to 42 mg DMT (approx. 0.1 to 0.6 mg/kg) are combined with harmine in a dose range of 17 to 280 mg, or harmaline in a range of 5 to 28 mg, respectively (Gable, 2007). The peak of the ayahuasca effect lies around 60 to 120 min after oral intake with a total expected duration of 4 h (Riba et al., 2003). There is no known LD_50_ and although overall medical risks of ayahuasca seem to be considerably low, some lethal cases after intake in humans were reported in the literature (Schlag et al., 2022; Houle et al., 2021; Heise and Brooks, 2017).

### MDMA

MDMA (*3,4-methyl​enedioxy​methamphetamine*) is a synthetic amphetamine derivative that is illicitly used and known worldwide under the name *Ecstasy* (Simmler and Liechti, 2018). Due to its euphoric, pro-social and empathogenic properties it is commonly described as entactogen, but like other synthetic phenylethylamines it can as well be classified as atypical psychedelic. It acts as selective agonist at serotonin receptors, directly stimulates the release of both serotonin and norepinephrine and inhibits the reuptake of these two neurotransmitters plus dopamine. The described serotonin 2A receptor agonism points to overlaps regarding phenomenological experiences with classic psychedelics. The threshold for subjective effects is around 50 mg while the full spectrum of drug effects is expected between 75 and 125 mg (approx. 1 to 1.7 mg/kg), lasting for a duration of 5 to 7 h (Simmler and Liechti, 2018). The LD_50_ in most animal species is around 100 to 300 mg/kg indicating an expected LD_50_ of 10 to 20 mg/kg in humans. There are multiple reports of lethal MDMA-intoxications in recreational settings, mostly due to dehydration and combined drug intoxications (Simmler and Liechti, 2018; Holze et al., 2020).

### Ketamine

Ketamine is a synthetic cyclohexanone derivative that is used as an anesthetic and is listed on the World Health Organizations list of essential medicines for this indication (WHO, 2021). Chemically, the molecule is racemic with possible configurations as (*R*)- and (*S*)-ketamine, the latter possessing a higher anesthetic potency (Kamp et al., 2020). It significantly differs from other anesthetics, as it rather increases haemodynamic parameters (Vankawala et al., 2021) and exerts dissociative effects, sometimes even including *out-of-body-experiences* (Muetzelfeld et al. 2008). It is also used in recreational settings, and it can be classified as an atypical psychedelic with distinctive psychological effects. Pharmacodynamically, it is known to inhibit glutamatergic N-methyl-D-aspartate (NMDA)-receptors in cortical regions and furthermore modulates GABA_A_ and opioid receptors. Due to its oral bioavailability of only 17%, its clinical forms of use are mostly intravenous, intramuscular, and intranasal, but also include oral, subcutaneous, and sublingual applications. The common dose for a psychedelic ketamine effect is around 35 mg i.v. (racemate approx. 0.5 mg/kg) and 18 mg i.v. (esketamine, approx. 0.25 mg/kg) as an infusion over a time course of about 40 min, with psychoactive effects lasting up to 90 to 120 min (Kamp et al., 2020; Peltoniemi et al., 2016; Muetzelfeldt et al., 2008). In animals, the LD_50_ is over 600 mg/kg, being equivalent to approx. 4 g in humans. In the literature, there are multiple reports of lethal intoxications in humans, mostly cases of combined drug intoxications with respiratory depressants in young males (Zanos et al., 2018; Hansen et al., 1988; Darke et al., 2021).

## Subjective effects

Psychedelic drugs have profound psychoactive effects, which are characteristic and reliably reproducible, and include alterations of perception, affectivity, consciousness and self, sometimes resulting in transpersonal or mystical experiences (Vollenweider and Preller, 2020; Nour and Carhart-Harris, 2017). At the same time, however, they are difficult to describe, due to their disparity from everyday conscious experience. Outside the medical framework the so-called *trip* is an overarching and popularly used term to characterize the inner journey people go through after taking a psychedelic (Bohling, 2017). Yet, the onset and experience of these altered states of consciousness are not only determined by the substance itself, but also by the psychological state of the individual taking the substance and the surroundings this happens in, illustrating two other popular terms: *set* and *setting* (Carhart-Harris et al., 2018). The comprehensive similarity between classic and atypical psychedelics is exactly this phenomenological *trip-*structure, whereas the temporal characteristics and details of the subjective experience may be distinctly different.

### Perception, etc.

On the 16th of April 1943 Albert Hofmann involuntarily came in physical contact with LSD, a substance with which he was working during his laboratory investigations into ergotamine derivates. The consequence was „a dreamlike state, with eyes closed, I perceived an uninterrupted stream of fantastic pictures, extraordinary shapes with intense, kaleidoscopic play of colors." (Hofmann, 1979, Hofmann, 2012), a beautiful first-hand description. Changes in sensory perception can reach from slight changes in visual and spatial perception to synesthesia and illusions with colorful moving geometric patterns and objects, up to complex scenic and dream-like episodes (Kometer and Vollenweider, 2018). The rich and diverse perceptual changes under the influence of psychedelics have inspired the work of generations of artists, musicians, and authors, sometimes referred to as *psychedelic art* (Masters and Houston, 1968).

### Affectivity

Psychedelic experiences are often accompanied by changes in mood (often euphoria) and emotional sensitivity, which in the right supportive setting may even lead to profound emotional release and breakthrough experiences (Preller and Vollenweider, 2018). Changes in emotion perception are seen as important mechanisms for the reported antidepressant effects of psychedelics (Breeksema et al., 2020). While with classic psychedelics, the reported experiences often emphasize on visual-aesthetic and transpersonal elements, the atypical psychedelic MDMA more typically induces a strong emotional-tactile form of stimulation (Holze et al., 2020). Intensive euphoria, feelings of peace and perfection and a desire for closeness and harmony are frequently reported. This can also be associated with a heightened desire for non-sexual body contact, feelings of affection and empathy for surrounding people and an increased wish to communicate (Sumnall et al., 2006). MDMA-induced reprocessing of traumatic memories and emotional engagement with therapeutic processes are also understood as core mechanisms, here for the treatment of post-traumatic stress disorder (PTSD) (Feduccia and Mithoefer, 2018).

### Consciousness and self

The consciousness itself including the perception of self and time can be deeply altered under psychedelics, sometimes resulting in new insights about oneself or a sense of oneness with the environment. In more intense forms, total ego-dissolution or a feeling of eternity may occur, sometimes described as mystical and transpersonal experiences (Vollenweider and Preller, 2020; Nour and Carhart-Harris, 2017). These effects explain why psychedelic drugs were and still are used in spiritual practices around the world, to transcend the structure of everyday reality and to connect to some form of higher entity (Baker, 2005). The occurrence of insights and the shifting from a rigid self-focus towards a stronger connectedness with the world and beyond, are also understood as important antidepressant mechanisms of psychedelics (Breeksema et al., 2020). Ketamine produces distinct dissociative alterations of the consciousness and the self, such as derealization and depersonalization (Muetzelfeldt et al., 2008). In higher doses, out-of-body experiences and characteristic forms of ego-dissolution may occur. In hedonistic user subcultures, this dissociation from normal body experience and sometimes total inability to communicate is referred to as *k-*hole (Muetzelfeldt et al., 2008)*.* Due to this derealization and depersonalization, ketamine produces a more pronounced feeling of immobilization than other psychedelics (Vlisides et al., 2018).

### Subjective experiences in clinical populations

A recent investigation on the subjective experiences of patients undergoing treatments with various psychedelics identified some overlapping phenomena of potential therapeutic significance (Breeksema et al., 2020). Fifteen studies were analyzed including 178 patients suffering from various psychiatric disorders including anxiety, depression, eating disorders, PTSD, and substance use disorders. Substances included psilocybin, LSD, ibogaine, ayahuasca, ketamine and MDMA. Subjective phenomena that patients experienced as clinically and personally important, were insights, altered self-perception, increased connectedness, transcendental experiences, and an expanded emotional spectrum (Breeksema et al., 2020).

### Psychological adverse effects

Under the influence of both classic and atypical psychedelics psychological complications and adverse effects may occur. Acute symptoms like disorientation, panic, fear and overwhelming distress—often referred to as *bad trip*—may arise, especially in a difficult setting (Johnson et al., 2008; Baylen and Rosenberg, 2006; Perry et al., 2007). In a study investigating negative experiences under psilocybin in 1993 recreational users, 39% rated it among the top five most challenging experiences of his/her life and 11% put self or others at risk of physical harm. However, 84% reported still having benefited from the experience (Carbonaro et al., 2016). Also, with MDMA bad experiences are reported especially in higher doses, for example in the form of alterations of judgement, overwhelming experiences, or feelings of total dissolution (Sumnall et al., 2006). Nevertheless, an investigation on potential long-term effects of psychedelics (LSD, psilocybin, mescaline, peyote) after non-medical use in 21′967 subjects did not show increased life-time risk for the occurrence of psychiatric disorders (Krebs and Johansen, 2013). One recent meta-analysis even found decreased suicidality following psychedelics administered in a therapeutic setting, which again emphasizes the importance of set and setting variables (Zeifman et al., 2022). Moreover, also large population-based studies found use of psychedelics associated with reduced psychological distress and suicidality (Hendricks et al., 2015; Jones and Nock, 2022b), depression (Jones and Nock, 2022a), lowered odds of crime arrests (Jones and Nock, 2022c; Hendricks et al., 2018), lowered odds of opiate use disorder (only psilocybin) (Jones et al., 2022), lower odds of heart disease and diabetes (Simonsson et al., 2021a), and lower odds of overweight (Simonsson et al., 2021b). However, it should be further noticed that psychedelics have an outstanding suggestive potency. Not only in non-medical users but also in patients, researchers, and clinicians, they may evoke strong expectation biases, increased suggestibility, and idealization. The latter is also illustrated by the increasing popularization of these substances among various groups (Yaden et al., 2021; Anderson et al., 2020).

## Mechanisms of action

### Neurobiological mechanisms

The resurgent interest in the psychopharmacological potential of psychedelics has led to a deepened understanding of their underlying neurobiological mechanisms. In various areas of interest from neurotransmission and receptor activation up to brain connectivity changes and network alterations, but also predictive processing and neuroplasticity, a tremendous amount evidence has been created (Vollenweider and Preller, 2020; McClure-Begley and Roth, 2022). Interestingly, there are important parallels to state-of-the-art antidepressants, where alterations in emotional processing, social interaction and neuroplasticity are also discussed as new therapeutic mechanisms (Harmer et al., 2017). Starting with the receptor level, all classic psychedelics act as agonists on cerebral serotonin 2A receptors, primarily on apical dendrites of pyramidal cells in layer V of the neocortex (Nichols, 2016). This basic mechanism seems crucial for the broad spectrum of behavioral and subjective effects of these drugs. Already in 1998, Vollenweider and colleagues described the fundamental principle of preventing psychedelic effects induced by psilocybin by simply blocking serotonin 2A receptors using the specific antagonist ketanserin (Vollenweider et al., 1998). A finding that was later reproduced for the psychedelic effects of DMT and LSD (Valle et al., 2016, Preller et al., 2017). The activation of serotonin 2A receptors leads to a downstream increase in cortical glutamate concentrations (Martin-Ruiz et al., 2001). Furthermore, dopaminergic neurotransmission is facilitated, as shown with psilocybin by increasing striatal dopamine release, which was associated with subjective euphoria and depersonalization (Vollenweider et al., 1999). Other neurotransmitter-modulations are diverse across substances, but a potential causal relationship between other neurotransmitters and characteristic classic psychedelic effects is still lacking.

Classic psychedelics modulate different neuronal networks that process sensory perception, the experience of self, and emotion regulation, which are relevant for the neurobiology of depression (Vollenweider and Kometer, 2010; Vollenweider and Preller, 2020). Several studies demonstrated classic psychedelics-induced decreases as well as increases of brain resting state functional connectivity of the default mode and the salience networks (Palhano-Fontes et al., 2015; Speth et al., 2016; Carhart-Harris et al., 2017; Muller et al., 2018; Pasquini et al., 2020). Brain areas of interest are the prefrontal cortex, the thalamus, the anterior and posterior cingulum, and the claustrum, converging in three core models of the neurobiological mechanisms of the psychedelic experience (Castelhano et al., 2021; Carhart-Harris and Friston, 2019; Vollenweider and Geyer, 2001). In the CSTC model (from in *cortico-striato-thalamo-cortical* [CSTC] feedback loops), psychedelic phenomena are explained by a reduction of the thalamic filter-function and a consecutive overload of prefrontal areas with internal and external information (Vollenweider and Geyer, 2001). In principle, this idea of the brain as a *reduction valve* during everyday conscious experience was already suggested by Aldous Huxley in his famous essay *The Doors of Perception* (Huxley, 1954). While the thalamic filter model builds on evidence of altered thalamic gating in CSTC feedback loops, newer imaging studies provide evidence for changes in sensory processing: connectivity between sensory cortices increases (potentially explaining the frequent synesthetic phenomena), while integration of associative cortical areas decreases (Vollenweider and Preller, 2020). The *relaxed beliefs under psychedelics *(REBUS) model postulates that the psychedelic brain shows increased neuronal entropy in the context of enhanced predictive processing (Carhart-Harris and Friston, 2019). This idea builds on the conception of the brain working under the premises of Bayesian inference, thus constantly generating, and updating prior beliefs of its own state and its environment. With reference to psychedelic states, the REBUS model suggests that prior beliefs are less constrained and less precise under the influence of psychedelics, potentially allowing a more intense modulation of top-down cognition by incoming bottom-up sensory stimuli. Keeping in mind that rigid beliefs and negative biases about oneself and the world are key features of depression, opening up these beliefs and biases for new perspectives might already be a mechanism for potential antidepressant effects of psychedelics (Carhart-Harris and Friston, 2019). A third model, which was partly derived from resting-state functional brain connectivity studies, postulates that characteristic psychedelic effects result from decreased control from top-down networks and an increase of the excitability of structures processing sensory, emotional and cognitive functions, which is reflected by reduced functional connectivity of and within default mode network (Castelhano et al., 2021). Here, the claustrum, a thin area of grey matter between the insula and the external capsule that densely expresses serotonin 2A receptors, may be the core structure of functional decoupling of top-down and bottom-up networks (Doss et al., 2022).

Furthermore, current studies in animal- and in-vitro-models provide evidence for mostly glutamate-induced increases in neuroplasticity, likely also initiated by serotonin 2A receptor activation (Aleksandrova and Phillips, 2021; de Vos et al., 2021). Here, brain-derived neurotrophic factor (BDNF) is one of the most important mediators of neuroplasticity under psychedelics (Vollenweider & Kometer, 2010). The involved processes are responsible for the regulation of synaptogenesis, learning and memory consolidation. Moreover, several studies also found evidence for anti-inflammatory properties of classic psychedelics (Nichols et al., 2017). These post-acute effects are seen as a central element of the long-lasting therapeutic effects of these drugs (Ly et al., 2018).

A multitude of receptor mechanisms and secondary processes is responsible for the effects of MDMA (Simmler and Liechti, 2018). It primarily promotes the release of serotonin, norepinephrine and to a lesser extent dopamine. The most important effects are explained by activation of the serotonergic system resulting in reduced amygdala-reactivity to adverse stimuli (Carhart-Harris et al., 2015). In addition, an increased release of the social-bonding hormone oxytocin is observed after the ingestion of MDMA, which partly explains the prosocial and empathogenic effects of the drug (Sessa et al., 2019).

Ketamine primarily acts as antagonist at glutamatergic NMDA-receptors, although a secondary activation of glutamatergic α-amino-3-hydroxy-5-methyl-4-isoxazolepropionic acid (AMPA)-receptors can be observed. Different brain areas are involved in the neurobiological processing of ketamine’s subjective effects, such as the prefrontal cortex, the subgenual anterior cingulate cortex, the hippocampus, and the reward system (Ionescu et al., 2018). Compelling evidence points to the central role of increased neuroplasticity for the antidepressant effects of ketamine, resulting from changes in the regulation of various growth factors including BDNF, eukaryotic elongation factor 2, mechanistic target of rapamycin and glycogen synthase kinase-3 (Zanos and Gould, 2018).

### Psychological mechanisms

After an interruption of almost 50 years, a renewed interest for the scientific investigation of psychedelics-induced psychological phenomena and their potential therapeutic effects in neuropsychiatric disorders has evolved, recently. Here, alterations in emotion-, self-, and social-processing have been identified as core mechanisms (Vollenweider and Preller, 2020).

#### Emotion-processing

Several human studies could show that classic psychedelics like LSD and psilocybin reduce affective and neurobiological reactions to negative stimuli (Kraehenmann et al., 2015, 2016; Mueller et al., 2017). For example, LSD (Dolder et al., 2016) and psilocybin (Kometer et al., 2012), impair the recognition of fearful face-expressions, which is accompanied by reduced amygdala reactivity. These changes even seem to be long-lasting, as altered emotion perception after a single dose of psilocybin endured for up to 1 month after administration in patients with treatment resistant unipolar depression (Stroud et al., 2018). Since a pronounced negative bias is seen as hallmark symptom and etiological factor of major depression, this mechanism could have counteracting effects and thus exert a therapeutic function. The adjustment of negative biases might allow patients to reflect on depressive thoughts and self-percepts with a less negative attitude and greater psychological flexibility (Wolff et al., 2020).

#### Consciousness- and self-processing

Altered experiences of the consciousness and the self are consistently reported under the influence of psychedelics (Nour and Carhart-Harris, 2017). These include altered self-perception, insights and catharsis, as well as changes in the self-to-other discrimination, to feelings of total ego-dissolution, and a mystical feeling of unity (Milliere et al., 2018). Psychedelics-induced mystical experiences were associated with positive psychological outcomes in healthy subjects and patient populations (Johnson et al., 2019). In an early study on LSD-assisted psychotherapy, insightful and cathartic experiences were accompanied by clinical improvement (Gasser et al., 2015). The feeling of awe, which is an emotion experienced in the presence of an overwhelming stimulus requiring an adaptation of mental constructs, is also discussed as an important mechanism in this context (Hendricks, 2018). As mentioned above, a comprehensive review on clinically important subjective phenomena under psychedelics reported insights, altered self-perception, increased connectedness, transcendental experiences, and an expanded emotional spectrum as some of the most significant experiences (Breeksema et al., 2020). It is important to notice that plenty of studies have shown a correlation between positively experienced self-dissolution or feeling of unity and therapeutic effects in addiction, anxiety, and affective disorders (Nour and Carhart-Harris, 2017; Roseman et al., 2017). However, it is still difficult to unambiguously attribute these effects either to the treatment per se or to expectation-enhanced placebo effects.

#### Social-processing

Additionally, current studies suggest psychedelics-induced alterations in social and interpersonal processing as important therapeutic mechanisms (Forstmann et al., 2020; Preller et al., 2018). Social withdrawal and impaired social cognition are ubiquitous in psychiatric disorders, as both causal and maintaining factors (Fett et al., 2015). Psychedelics like psilocybin interfere with these relevant mechanisms by reducing negative feelings of social isolation and rejection, and potentially reinstating emotional empathy and social reward processes (Preller et al., 2016). After one-time administration of psilocybin, patients suffering from depression reported more positive connection to their social environment, which was reported as one of the most important factors contributing to their treatment success (Watts et al., 2017).

#### Mindfulness and acceptance

In recent psychotherapy approaches, especially of the so-called third wave, mindfulness and acceptance are important therapeutic strategies for the management of psychosocial distress (Khoury et al., 2015), and there is growing evidence that mindfulness techniques and psychedelics have synergistic positive effects (Heuschkel and Kuypers, 2020). Recent models aim at integrating increased psychological flexibility and acceptance into the framework of psychedelics-assisted psychotherapy (Watts and Luoma, 2020; Wolff et al., 2022). In a study with 39 experienced meditators receiving a single dose of 0.315 mg/kg psilocybin p.o. in a placebo-controlled design, a significant positive association between psychedelic effects and mindfulness was observed (Smigielski et al., 2019). Psilocybin led to a deepening of meditative states and more intensive positively experienced self-dissolution. In the 4-months-follow-up, subjects in the psilocybin group still reported significantly higher mindfulness ratings and positive alterations in psychosocial functioning compared to placebo (Smigielski et al., 2019). Critically reflecting the data, it is important to mention that studies have not shown additional clinical efficacy of mindfulness augmentation of classic cognitive behavioral psychotherapy, yet (Cuijpers et al., 2019).

#### Set and setting in the psychedelics-assisted psychotherapy

The variables of *set and setting* were recognized as crucial therapeutic factors, already in the early phase of the psychiatric-psychotherapeutic use of psychedelics (Gukasyan and Nayak, 2021). Here, it is important to mention that expectancy effects may have a strong idealistic or spiritual undertone in some subcultures (Lieberman, 2021). Early psychotherapeutic use of psychedelics was orientated at the psychoanalytic methods of its time and aimed at releasing subconscious and repressed psychic material to be integrated in the therapeutic process (Nichols and Walter, 2021). This was also mentioned in the information material coming with Sandoz pharmaceutical LSD called Delysid (Sandoz, 1964). In this framework, the therapeutic effect is not primarily attributed to the substance itself but rather to its capacity to cathartically release blocked emotions and memories, which are then used as therapeutic material (Harris, 2021). Here, the early German psychedelic pioneer Hanscarl Leuner should be mentioned, who used his own therapeutic experiences with psychedelic substances to develop his psychotherapeutic technique of Guided Affective Imagery (Leuner, 1969). Current therapeutic frameworks also emphasize on the elements of set, setting and psychotherapeutic integration and implement these elements into new treatment paradigms using classic and atypical psychedelics (Nutt and Carhart-Harris, 2021; Holtzheimer and Mayberg, 2011; Dore et al., 2019). The result is a concept of psychedelic-assisted psychotherapy in which sporadic and professionally guided substance experiences should be integrated into a continuous psychotherapy, with a not yet defined duration and frequency of sessions.

#### Ketamine controversy

Both neurobiological and conceptional differences between the therapeutic principles of classic psychedelics and MDMA on the one, and ketamine on the other hand can be drawn. The use of ketamine and esketamine in its intravenous and intranasal solutions initially followed the traditional approach of substances acting via molecular mechanisms and not necessarily by producing altered subjective experiences. Indeed, investigations show that conscious phenomenological changes appear not to be a necessary condition for the antidepressant response to ketamine (Ballard and Zarate, 2020). However, also ketamine-induced psychedelic experiences might be of additional therapeutic value, when used in a psychedelic-assisted psychotherapy context (Mathai et al.,^2020^, ^2022^; Mollaahmetoglu et al., 2021; Dore et al., 2019).

### Psychedelics in the treatment of unipolar and bipolar depression

Several studies investigating the antidepressant effects of classic psychedelics including LSD, psilocybin and mescaline were performed in the 1950s to 1970s. These studies did not typically follow a double-blind and placebo-controlled design, thus the overall efficacy in reducing depressive symptoms by 80% in a total of over 420 patients should be interpreted carefully, rather as a positive signal, than as robust clinical evidence (Rucker et al., 2016). The international ban on psychedelics also ended clinical research with these molecules, until the current revival in the early 2000s (Griffiths et al., 2006). Soon also clinical studies using psychedelics in psychiatric conditions with affective symptoms were initiated. In one of the first such investigations, the effect of a single dose of psilocybin (0.3 mg/kg p.o.) as an adjunct to psychotherapy was tested on anxiety and depression in 29 patients with cancer. In this double-blind study with an active placebo-controlled crossover design using niacin, psilocybin led to a clinically significant symptom reduction, which was still measurable in 60–80% of the patients at the 6.5-month follow-up. The authors identified the psilocybin-induced mystical experience as a main mediator of the therapeutic effect (Ross et al., 2016). Weaknesses of the study were functional unblinding due to obvious psychedelic effects (although an active placebo was used) and a small and non-representative sample, both of which are typical for this phase of clinical psychedelic studies. In another study with 51 terminally ill cancer patients, a high dose of psilocybin (22 or 30 mg p.o.) compared to a placebo-like low dose of psilocybin (1 or 3 mg p.o.) also led to a decrease of depressed mood and anxiety, with about 80% of patients continuing to show improvements at a 6-month follow-up (Griffiths et al., 2016). A first open-label feasibility trial in 12 patients with treatment-resistant unipolar depression using two doses of psilocybin (10 mg and 25 mg p.o.) combined with psychological support showed promising results. Adverse effects were all transient and included initial anxiety, confusion and thought disorder, nausea, and headache, while symptom reduction was rapid and sustained up to 3 months after high-dose treatment (Carhart-Harris et al., 2016).

Observational and experimental studies in healthy volunteers offered indications that also ayahuasca might have antidepressant properties (Dos Santos et al., 2016), which was confirmed in a first small open-label study with six depressive patients (Osorio Fde et al., 2015). In a following open-label study with a larger sample of 17 patients with recurrent major depression, a single dose of ayahuasca had a significant and rapid antidepressant effect, which lasted for 3 weeks (Sanches et al., 2016). The ayahuasca potion contained 0.8 mg/ml DMT, 0.21 mg/ml harmine, and no harmaline, and patients received 2.2 ml/kg, resulting in individual doses of 96 to 160 mg of DMT and 25 to 42 mg of harmine. Psychedelic effects of the drug were mild, and vomiting was the most common adverse effect (47%). A weakness of this study was that the patients mostly suffered from mild and moderate depression (only one severely depressed patient) (Sanches et al., 2016).

During this first wave of new open-label clinical investigations with psychedelics, also LSD was shown to have beneficial effects in patients with psychiatric symptoms. In 12 patients with life-threatening diseases, two sessions with 200 µg and 20 µg LSD p.o. respectively, which were integrated into psychotherapy, led to a sustained reduction of anxiety (Gasser et al., 2014). Given the standard caveats of psychedelic studies such as functional unblinding and positive expectancy effects (Lieberman, 2021), these uncontrolled studies should be interpreted with caution, especially as the sample sizes were very small. Still, these were important preliminary investigations, which added evidence to the promising results of other early studies and inspired further investigations with more rigorous designs.

Unfortunatley, systematic studies are still limited to unipolar depressed patients, as patients with bipolar disorder have been excluded from recent psychedelic trials due to safety concerns. These concerns stem mostly from qualitative user reports or case studies, which documented the occurrence of switches to mania after ingestion of psychedelics (see Table 1). One case study of a 21-year-old woman described the occurrence of psychotic mania about 36 h after a single ingestion of psilocybin-containing mushrooms, which could be stabilized first with lithium and aripiprazole, and which was later successfully switched to lamotrigine due to adverse effects. The patient had a positive family history of bipolar disorder (father and paternal grandmother) and was suffering depression and PTSD, but was unmedicated at the time of the episode (Hendin and Penn, 2021). Ayahuasca and DMT were also reported to induce mania with psychotic features in patients with bipolar disorder or a positive family history of bipolar disorder. A 40-year-old male psychiatrist with known bipolar disorder (a single previous manic episode) was hospitalized with mania and psychosis after self-medicating for depression. He took up to 1 g daily of vaporized DMT for 6 months and then added the MAO inhibitor phenelzine (60 mg p.o.) 3 weeks before the hospitalization. A combination of lithium 1200 mg/d, paliperidone 6 mg/d, and clonazepam 3.5 mg/d as sleep aid led to stabilization, but a follow-up was not possible (Brown et al., 2017b). Another 25-year-old male with known bipolar disorder and history of cannabis abuse, was hospitalized with mania with psychotic features, which occurred 2 days after ayahuasca ingestion. Remission was achieved with a combination of benperidole, olanzapine and lorazepam (Zellner et al., 2019). A 30-year-old male with previous hypomanic episodes and a positive first-degree family history of bipolar disorder developed mania with psychotic features, also 2 days after a ayahuasca ritual, which could be stabilized with risperidone 2 mg/d and clonazepam 2 mg/d (Szmulewicz et al., 2015). The etiological classification of ayahuasca-related mania is especially difficult, as the drug consists of a classic psychedelic (DMT) and a MAO inhibitor (harmine and harmaline). MAO inhibitors are—like other antidepressants—known to induce manic switches in patients with unipolar depression (Wada et al., 2006). Moreover, psychosis with schizophrenic features such as hallucinations and paranoid delusions, rather than mania, was reported to be a rare but possible adverse reaction to ayahuasca or DMT use in a systematic review of existing case reports (Dos Santos et al., 2017). LSD was reported to have induced a first-time manic episode with psychotic features after 3 weeks post ingestion, which could be stabilized with lithium treatment (Lake et al., 1981).

**Table 1:** Overview of case reports of psychedelics-induced mania

Clinical case	References
• 21-year-old woman • Depression and PTSD, unmedicated • Positive family history of bipolar disorder (father and paternal grandmother) • Psychotic mania 36 h after ingestion of psilocybin-containing mushrooms • Stabilized with lithium and aripiprazole, later lamotrigine	Hendin and Penn, 2021
• 40-year-old male psychiatrist • Known bipolar disorder (a single previous manic episode) • Hospitalized with psychotic mania after self-medicating for depression: 1 g daily of vaporized DMT for 6 months, then plus phenelzine (60 mg p.o.) 3 weeks before the episode • Stabilization with lithium 1200 mg/d, paliperidone 6 mg/d, and clonazepam 3.5 mg/d • No follow-up possible	Brown et al., 2017b
• 25-year-old male • Known bipolar disorder and history of cannabis abuse • Hospitalized with psychotic mania 2 days after ayahuasca ingestion • Remission with benperidole, olanzapine and lorazepam	Zellner et al., 2019
• 30-year-old male • Previous hypomanic episodes • Positive first-degree family history of bipolar disorder • Psychotic mania two days after a ayahuasca ritual • Stabilization with risperidone 2 mg/d and clonazepam 2 mg/d	Szmulewicz et al., 2015

First, this is an important safety issue, which must be taken into consideration for the design of studies or treatment protocols. Efficient screening procedures regarding individual or family history of bipolar disorders should be included. Moreover, a rigorous monitoring and follow-up of the occurrence of manic and/or psychotic symptoms following psychedelic use should help to identify if these substances pose safety concerns for populations with affective disorders. Furthermore, as gathering of data of psychedelics in patients with bipolar disorder is difficult under current conditions, it is important to investigate if an increased switch-to-mania rate can be detected in the studies with unipolar depressed patients. A recent ongoing study is investigating the safety, tolerability, and feasibility of psilocybin therapy in people with bipolar II disorder (NCT05065294, 2022). Second, the reported switches-to-mania might also be an indicator for antidepressant efficacy, as most antidepressant drugs are known to induce mania in a low but significant proportion of patients with depression (Viktorin et al., 2014). It is important to note, that the described cases of psychedelics-related mania all included psychotic features, which is more rarely the case with antidepressant-related mania (Stoll et al., 1994). Strikingly, data from user websites also indicate an association of adverse events when psychedelics are co-administered with lithium. In fact, lithium in combination with a classic psychedelic was associated with the highest number of negative consequences including seizures (46.8%) and bad trips (17.7%) (n = 62 reports), while no seizures or bad trips were reported in combination with lamotrigine (n = 34 reports) (Nayak et al., 2021).

For unipolar depression-studies a four-step treatment approach has been developed: 1. assessment, 2. preparation, 3. experience and 4. integration (Nutt and Carhart-Harris, 2021). In the first step the psychological and somatic eligibility is thoroughly assessed. In this process, a history of psychotic or bipolar disorder in patients or first-degree relatives is widely considered as exclusion criterion. Patients with relevant somatic impairments such as uncontrolled or severe arterial hypertonia are also excluded from participation. Serotonergic medication such as selective serotonin inhibitors often needs to be tapered down with special attention given to possible discontinuation syndromes. Especially for ayahuasca, which includes MAO-inhibiting harmine and harmaline, a combination with serotonergic drugs is contraindicated. The second step aims to prepare the participants by explaining the range of possible effects and potential challenges and setting the right attitude for the psychedelic experience. Any potential concerns are addressed and a trusting relationship to the therapist and to the study environment should be established. The experience itself is then taking place in a comfortable and safe living-room-like setting, usually with the option to listen to relaxing music and to use blindfolds. The patient is invited to self-center, close the eyes, and observe the unfolding experience. During the experience the therapist is usually not actively interfering but remains in a supportive and if necessary, reassuring role. Already on the following day, the first post-experience integration session is conducted with the same therapist. The experiences and potential insights are gathered, integrated, and worked with in the further therapeutic process. Usually, more sessions follow to process the experience over time and provide integration into everyday life with the aim of finally facilitating lasting psychological and behavioral changes.

After the above mentioned first explorative and open-label studies were performed successfully, three more robust classic psychedelics treatment studies with more rigorous, randomized, placebo-controlled designs in unipolar depression followed, one with ayahuasca and two with psilocybin (Carhart-Harris et al., 2021; Davis et al., 2021; Palhano-Fontes et al., 2019). Ayahuasca showed rapid antidepressant effects in treatment-resistant depression in a recent Brazilian study (Palhano-Fontes et al., 2019). The ayahuasca potion was applied in ml/kg, which corresponded to doses of 0.36 mg/kg DMT, 1.86 mg/kg harmine, 0.24 mg/kg harmaline, and 1.2 mg/kg tetrahydroharmine, and was compared to an active placebo of comparable color and taste including zink sulfate, which induced low to modest gastrointestinal distress. The study was conducted using a parallel-arm (14 ayahuasca and 15 placebo), double-blind, randomized, placebo-controlled design in patients with treatment-resistant depression, but also 76% with comorbid personality disorders. The response rates were high for the ayahuasca and the placebo groups on days one and two, and were significantly higher for ayahuasca on day 7, while the between-groups remission rate showed only a trend towards significance on day seven (Palhano-Fontes et al., 2019). A strength of the study was the use of an active placebo with some ayahuasca-like adverse effects such as nausea and anxiety, but this probably also led to higher response rates in the placebo group. Even more so, as the sample included a high percentage of comorbid personality disorders (most of the cluster B), which tend to present higher placebo responses (Ripoll, 2013). Here, a lager sample size would certainly have helped to produce clearer results. Moreover, no considerable follow-up was conducted to assess effects posterior to the seventh day time point.

A randomized clinical trial investigating the effects of two psilocybin sessions (session 1: 0.28 mg/kg; session 2: 0.42 mg/kg) combined with supportive psychotherapy compared to a delayed-treatment waiting list in 27 patients with treatment-resistant depression, showed a response in 71% of the patients at weeks one and four, and remission in 58% at week one and in 54% at week four (adjusted for the delay period) (Davis et al., 2021). The delayed treatment waiting list design is an interesting method to reduce the effect of functional unblinding in studies with psychedelics. Still, expectancy effects cannot be ruled out with this approach. Other weaknesses of this study were the small sample size and a short-term follow-up period.

For psilocybin, a recent phase-2-study with 59 patients with moderate to severe unipolar depression showed a similar—but not greater—efficacy of the substance compared to the SSRI escitalopram over a duration of 6 weeks (Carhart-Harris et al., 2021). In this double-blind, randomized, controlled trial, 30 patients received two separate doses of 25 mg psilocybin 3 weeks apart plus 6 weeks of daily placebo (psilocybin group) and 29 patients received two separate doses of 1 mg psilocybin 3 weeks apart plus 6 weeks of daily escitalopram (escitalopram group), while all patients received psychological support. Comparisons of the changes of depression scores, response and remission rates at week 6 showed only trend-wise superiority of psilocybin compared to escitalopram (Carhart-Harris et al., 2021). Until now, this rigorous study provides the qualitatively highest standing evidence on the antidepressant properties of psilocybin. Although it does not prove a superiority on the primary endpoint, it does overall show an at least equal antidepressant effect along with substantially less pharmacological exposition within the 6 weeks of the trial (daily administration of SSRI compared to two psilocybin sessions). Nevertheless, the study has considerable limitations such as being underpowered (potential for false negative results), and that the improvement in the escitalopram group was not even on a standard placebo level. Moreover, standard psychedelics studies limitations like functional unblinding and expectancy effects for specifically selected (and highly interested in psychedelics) patients who are mostly recruited via advertisement do persist (Carhart-Harris and Friston, 2019, Lieberman, 2021). This can only be addressed in head-to-head comparisons in further robust studies with larger and independent study populations. Another potential challenge in this treatment paradigm is the intensity in terms of time and therapeutic resources as a guided psilocybin session requires around 6 h of therapist presence. Should psychedelic assisted psychotherapy be made available for larger parts of the population it is crucial to provide a reliable framework for the role of medical doctors, therapist training and supervision and an overall appropriate and safe application of these substances (Nutt and Carhart-Harris, 2021). Further studies will hopefully help to elaborate how populations with diverging attitudes and vulnerabilities may react to these potentially very intense and challenging experiences. This is of particular importance regarding patients with bipolar depression, as classic psychedelics seem to be associated with the induction of manic switches and, in combination with lithium, of epileptic seizures and bad trips (Lake et al., 1981; Hendin and Penn, 2021; Szmulewicz et al., 2015; Viktorin et al., 2014). Careful precautions are needed to prevent people from misuse and to establish reliable and professionalized medical procedures for the use of these promising substances.

In conclusion, the current outcome of studies with classic psychedelics in patients with unipolar depression is very promising, but not yet definitive. Further studies with larger sample sizes are warranted to establish clinical efficiency and later, adjustments in legal and therapeutic frameworks will be necessary conditions for an approval of these substances in major depression.

MDMA-assisted psychotherapy is currently not investigated in the treatment of unipolar or bipolar depression, but promising evidence was produced for the treatment of PTSD. A recent phase-3-study showed good tolerability and efficacy in 90 patients with severe PTSD (Mitchell et al., 2021). These encouraging results for MDMA in PTSD patients led to a designation as *breakthrough therapy* granted by the US Food and Drug Administration, a status that was recently also granted to psilocybin in the treatment of depression.

The antidepressant properties of ketamine have first been published in the year 2000 and have since then been established in a considerable amount of randomized controlled trials and meta-analyses (Berman et al., 2000; Bahji et al., 2021; McIntyre et al., 2020). These studies show that both the initially investigated intravenous use, but also the intranasal application of ketamine and esketamine show rapid therapeutic effects in unipolar depression. However, a recent meta-analysis suggests a potentially stronger effect of i.v. applications, although potential placebo and expectancy effects of this route of application are discussed (Bahji et al., 2021). For both ketamine i.v. and esketamine i.n. an antisuicidal effect was confirmed in a recent meta-analysis (Xiong et al., 2021). Subsequently, ketamine is currently being included in international treatment guidelines as an augmentation therapy for treatment-resistant unipolar depression (Swainson et al., 2021). Moreover, a recent Cochrane analysis also showed limited but existing evidence for antidepressant effects of single ketamine infusions in combination with mood stabilizers in bipolar depression (Dean et al., 2021). Further larger studies are needed to determine the safety profile and therapeutic effects of ketamine and esketamine in these patients.

## Conclusions

Since outcomes of currently available antidepressant treatments are limited, and parts of the pharmaceutical industry have withdrawn from psychopharmacological drug development, the demand for innovative therapies is high and growing. Here, psychedelics and psychedelic-assisted psychotherapy offer new and promising approaches for research and clinical treatments in psychiatry. Despite limitations like functional unblinding and expectancy effects, a growing number of small and medium sized studies show encouraging effects in psychiatric disorders such as unipolar depression and PTSD. Investigating classic psychedelics in patients with bipolar disorder is not yet recommended, as an association with switches to mania has been reported in case studies. Therefore, the investigation and monitoring of the occurrence of manic symptoms should be a standard practice in all psychedelic studies in clinical populations.

At the same time, an increasing popularization of psychedelics is observable, with obvious parallels to the 1960s including idealization in certain subcultures and spreading uncontrolled use. Here, the urgency of a strictly scientific, thoughtful, and professional clinical practice comes into play, again. It will depend on some crucial factors, whether the current renaissance of psychedelic substances will be fruitful and sustainable on a broader societal level. One important factor will be the implementation of strict ethical and practical standards, controlled and legitimized by the professional medical societies through transparent and peer-reviewed processes (Anderson et al., 2020; Petranker et al., 2020). Further, longitudinal studies with larger sample sizes und independent study populations will be needed to establish the therapeutic efficacy of psychedelics in psychiatric conditions (Vollenweider and Preller, 2020). Under these premises, an integration of these promising and fascinating substances into contemporary biomedicine seems feasible and even desirable.

## Data Availability

Not applicable.

## Abbreviations

AMPA: α-Amino-3-hydroxy-5-methyl-4-isoxazolepropionic acid
BDNF: Brain-derived neurotrophic factor
CSTC: Cortico-striato-thalamo-cortical
DMT: N,N-dimethyltryptamine
LSD: Lysergic acid diethylamide
MAO: Monoaminoxidase inhibitors
MDMA: 3,4-Methyl​enedioxy​methamphetamine
NMDA: N-methyl-D-aspartate
PTSD: Post-traumatic stress disorder
REBUS: Relaxed beliefs under psychedelics
